# Near-field ptychography: phase retrieval for inline holography using a structured illumination

**DOI:** 10.1038/srep01927

**Published:** 2013-05-31

**Authors:** Marco Stockmar, Peter Cloetens, Irene Zanette, Bjoern Enders, Martin Dierolf, Franz Pfeiffer, Pierre Thibault

**Affiliations:** 1 Department of Physics and Institute for Medical Engineering, Technische Universität München, 85748 Garching, Germany; 2 European Synchrotron Radiation Facility (ESRF), 38000 Grenoble, France

## Abstract

Inline holography is a common phase-contrast imaging method which uses free-space propagation to encode the phase signal into measured intensities. However, quantitative retrieval of the sample's image remains challenging, imposing constraints on the nature of the sample or on the propagation distance. Here, we present a way of simultaneously retrieving the sample's complex-valued transmission function and the incident illumination function from near-field diffraction patterns. The procedure relies on the measurement diversity created by lateral translations of the sample with respect to a structured illumination. The reconstruction approach, in essence identical to that employed in ptychography, is applied to hard X-ray synchrotron measurements and to simulations. Compared to other inline holography techniques, we expect near-field ptychography to reduce reconstruction artefacts by factoring out wavefront imperfections and relaxing constraints on the sample's scattering properties, thus ultimately improving the robustness of propagation-based X-ray phase tomography.

X-ray phase contrast is a powerful imaging modality nowadays commonly used to produce quantitative maps of weakly absorbing objects. The method is used in biomedical research^1^,^2^, materials science^3^, and palaeontology^4^ among others. All phase-contrast imaging techniques are based on means to transform the phase shift induced by a sample on the incident wave into a measurable intensity signal. How the phase is retrieved from the measured intensity depends heavily on the nature of the recorded signal.

In its simplest form, inline holography encodes the phase through interference of the scattered wave with the co-propagating illumination^5^,^6^,^7^,^8^. For short enough effective propagation distances, the technique reveals directly in the measured intensity the contours of samples that produce phase shifts in the incoming wave – an effect especially useful to make visible weakly absorbing objects. While this so-called propagation-based phase contrast is sometimes sufficient for visualizing a sample's features, many imaging applications require the phase shift to be recovered quantitatively from the measured hologram.

The earliest phase retrieval approach, proposed by Gabor^9^, consists in back-propagating the measured intensity physically or computationally. To avoid the ensuing twin-image problem, more advanced techniques have been developed for short^7^ or multiple^10^ propagation distances, or for samples that are weakly scattering or made mostly of a known chemical composition^11^. Most of these techniques are based on linearisation, either of the propagation operator or of the sample transmission function, thus only valid for short propagation distances or weakly scattering objects respectively.

Other paths to phase retrieval involve experimental devices where the incoming beam is prepared to produce well-controlled interference, either through crystal diffraction^12^,^13^,^14^ or with gratings^15^,^16^. Although these techniques do not necessarily scale to higher resolutions, they benefit greatly from the diversity provided by multiple measurements to decouple the patterned wavefront from the object transmission function. Nevertheless, the incident wavefront remains a limitation as long as it must be known *a priori*. Speckle tracking techniques introduced recently^17^,^18^ can be used to perform phase retrieval without prior knowledge of the disturbed wavefront, though at the price of a decreased spatial resolution.

In this article we present an approach to solve the phase problem in the near-field regime, by which a sequence of diffraction patterns is collected for different lateral translations of the sample with respect to an unknown illumination. The technique applies to high Fresnel number diffraction, *i.e.*, *W*^2^ ≫ λ*z*, where *W* is the lateral extent of the illuminated area, λ is the wavelength and *z* is the effective propagation distance. To ensure sufficient diversity in the measurement and to provide contrast enhancement, the incident illumination must be static and deviate strongly from a uniform plane wave.

The reconstruction problem entails retrieving the sample's transmission function *T*(**r**) and the incident illumination *ψ*(**r**) given a set of intensity measurements *I_j_*(**r**) defined by 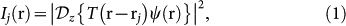
where 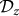 represents free-space propagation over a distance *z* and **r***_j_* is the lateral displacement of the sample relative to the illumination for the *j*th intensity measurement. Both *T*(**r**) and *ψ*(**r**) are two-dimensional complex-valued entities. For short effective propagation distances, corresponding to Fresnel numbers much larger than unity, the propagator 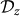 is well-behaved numerically^19^ when it is computed as a sequence of two fast Fourier transforms: 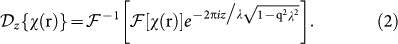
In this expression 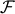 denotes the Fourier transform operation with reciprocal coordinate **q**. This specific implementation of the wavefield propagator is sometimes called the “angular spectrum method”^19^ since the two-dimensional Fourier transform of the wave, 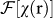, gives precisely the spectrum of the wave mapped on the Ewald sphere.

The solution to Eq. (1) can be retrieved using the algorithmic tools recently developed for an imaging technique called ptychography. Ptychography was originally described in the context of electron microscopy^20^ but more recently commonly used with X-rays^21^,^22^,^23^,^24^. The experimental procedure entails combining multiple diffraction measurements collected as a sample is scanned through a localized illumination, usually called the probe. A ptychographic dataset is collected in such a way that information is sufficient to reconstruct simultaneously the image of the sample and the illumination function. Various iterative algorithms^25^,^26^,^27^,^28^ have been devised to accomplish this task. Reconstructions shown in this paper are done using an implementation of the difference map^26^.

Previous experiments have successfully combined coherent diffractive imaging methods with Fresnel diffraction, most notably “keyhole imaging”^29^ and “Fresnel ptychographic coherent diffractive imaging”^30^. The approach proposed here differs from this earlier work by the fact that the illumination is extended over most of the reconstruction field of view, thus leading to Fresnel numbers that are orders of magnitudes larger, and leading to the violation of the oversampling condition usually required in all coherent diffractive imaging techniques. As a result, the resolution is determined, as in projection microscopy, by the demagnified detector pixel size and the source size, and not by the angular opening subtended by the detector^6^,^8^. Unlike Fresnel or far-field ptychography, full-field illumination conditions make near-field ptychography readily applicable to currently existing propagation-based phase-contrast setups. As such, larger fields of view can be imaged with just a few measurements and weaker requirements on the detector's dynamic range. Unlike any other holography technique, our approach automatically removes artefacts coming from imperfections in the incident wavefield since the incident illumination is retrieved directly alongside with the object.

Ensuring that the system of equations (1) admits a unique solution (within noise limits) depends on multiple experimental factors. As usual with holography it is required that at least one Fresnel zone is resolved^8^. Another requirement, this one quite unlike classical inline holography, is that the incoming illumination differs strongly from a uniform wavefront. More precisely, sufficient diversity is present in the measurements if distortions of the Fresnel diffraction patterns are observable. Thus, the spatial frequencies of the incident wavefront should have a non-negligible amplitude on the typical scale of the diffraction fringes, given again by the extent of the first Fresnel zone. Because no *a priori* knowledge of the illumination is required, producing a structured illumination is in practice extremely simple. As shown below, imperfections in the X-ray optics can create enough disturbances, although a stronger diffuser, such as a piece of paper, is more suitable.

The scanning points **r***_j_* are chosen far apart enough to guarantee that all diffraction measurements differ strongly – an essential condition for robust reconstruction. Unlike far-field ptychography, which requires a relatively small and isolated illumination because of sampling constraints, near-field ptychography works even if a wide sample area is illuminated at once. As a result, much fewer diffraction measurements suffice for a successful reconstruction. If the probe and object are described by arrays of *N* complex numbers, then there are 4*N* real unknowns. Thus more than four Fresnel diffraction patterns are required to make the problem over-constrained. In practice we have found that a few more (we use 16) are needed to ensure the stability of the reconstruction algorithm, especially if the features in either the illumination or the sample image are sparse.

## Results

**Figure 1:**
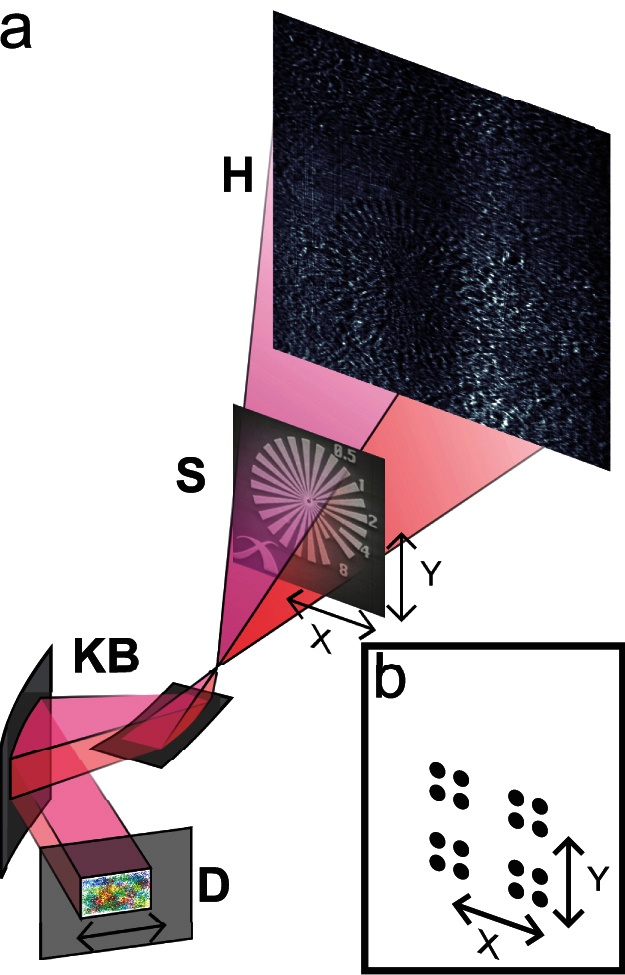
Schematic of the experimental setup used for X-ray near-field ptychography. (a) X-rays from an undulator source (not depicted) are focussed by Kirkpatrick-Baez mirrors (KB) to create a virtual point source and an expanding cone beam illumination to record a magnified near-field Fresnel diffraction pattern (H) of the sample (S). A stationary diffuser consisting of a piece of paper can be placed into the beam to modify the wavefront. The sample mounted on a high-precision stage is moved as depicted in (b) transversally.

First experiments were carried out at the nano-imaging endstation of beamline ID22 at the European Synchrotron Radiation Facility (ESRF) in Grenoble, France. The setup, shown in Fig. 1, has the usual configuration for holo-tomography normally conducted at this beamline^31^ with the exception of a static diffuser which can be inserted in the incident beam. A test pattern featuring a 30 μm diameter Siemens star, is placed in the beam expanding from the focus of a pair of Kirkpatrick-Baez mirrors. With appropriate rescaling of the detector pixel size and propagation distance, this cone beam inline holography configuration is known to be equivalent to a parallel beam configuration^32^,^33^.

**Figure 2:**
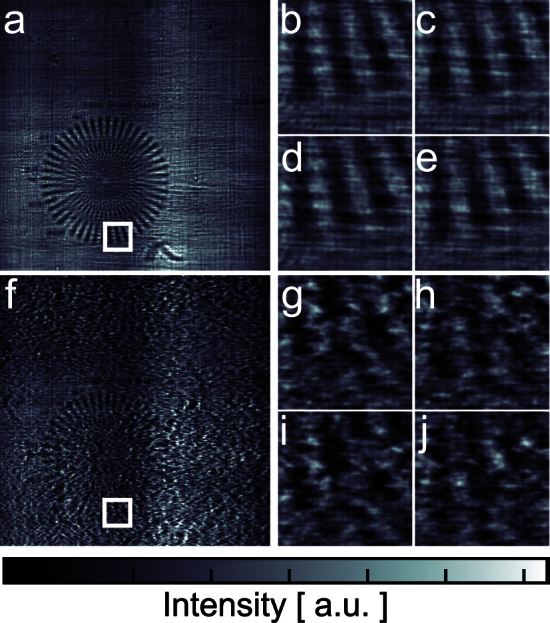
Near-field Fresnel diffraction patterns. (a) one of the 16 diffraction patterns recorded without diffuser. The test sample is easily recognizable despite illumination inhomogeneities coming from the beamline optics. (f) diffraction pattern of the same area, when the diffuser is inserted in the beam. Enlarged details of the diffraction patterns covering the same region of the sample (depicted by the white box in (a) and (f)) for four different illumination positions are shown in (b–e) and (g–j). Larger variations between the images are observed in the dataset taken with the diffuser.

A complete scan is made of 16 Fresnel diffraction patterns recorded for different transverse shifts of the sample with respect to the incident illumination. A selection of measured diffraction patterns is shown in Fig. 2 for two different illumination conditions. Figures 2(a) and 2(f) show one of the collected Fresnel diffraction patterns, respectively without and with the diffuser. These images highlight a fundamental difficulty with traditional holography data analysis, where removal of the incident wave fluctuations is normally accomplished through division by a flat field, *i.e.*, an image without sample. Such a procedure does not take into account the phase variations of the incident wave co-propagating with the sample transmission function and leads to inconsistency in the Fresnel diffraction patterns^32^. As a result, much effort is put in optimizing the X-ray optical properties of a setup to minimize wavefront distortions.

**Figure 3:**
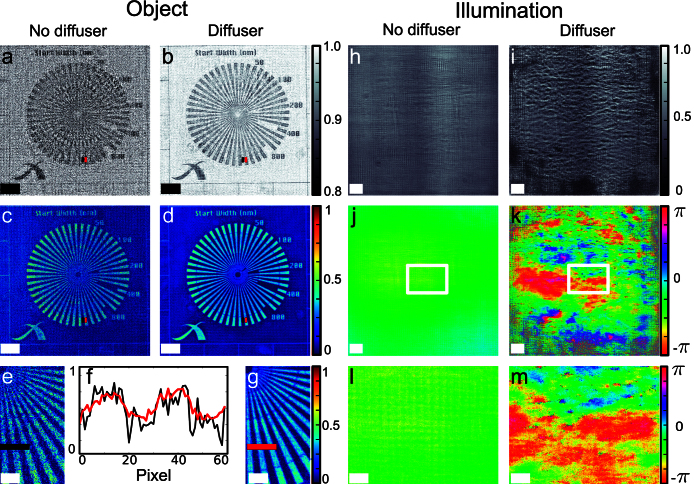
Reconstruction results. (a) and (c), modulus and phase of the reconstructed object using no diffuser. (b) and (d), same when the diffuser is present. (e) and (g), zoom into the phase image without and with diffuser respectively, the line plot, (f), shows the reduction of noise in the reconstruction when using a diffuser. On the right, reconstructed modulus, (h), and phase, (j), of the illumination without diffuser and (i) and (k), respectively with diffuser. (l) and (m), zoom into the phase to outline the structure. The scale bars indicate 5 μm and 2 μm for the magnified regions of interest. The red and black boxes in (a–d) indicate the ROIs used for the calculation of the CNR. Note that surrounding empty areas of the object were cropped.

The reconstructions are presented in Fig. 3. While phase retrieval is successful in both cases, the object transmission function (shown on the left hand side) has lower high frequency noise for the data collected with a diffuser. Computing the contrast-to-noise ratio (CNR) for a region of interest (ROI) as indicated in Fig. 3 yields values of 5.4 and 2.1 for the phase images with and without diffuser respectively. The phase part of both reconstructions has higher contrast than the amplitude, as is almost always the case with high-energy X-rays since the real part of the refraction index decrement is about eight times larger than its imaginary part (for the sample presented here – this ratio can reach three orders of magnitude with lighter elements^34^). The amplitude CNR computed with the same ROI is 0.44 and 0.26 for the datasets with and without diffuser respectively.

The higher quality of the reconstruction from the diffuser dataset should not come as a surprise. In a far-field geometry, strong fluctuations in the incident wavefield have been shown recently to improve significantly the signal-to-noise ratio^35^ and even to permit superresolution^36^. For full-field ptychography, the role of the diffuser is even more critical as it is the very source of diversity in the diffraction patterns – the lateral displacement of a perfectly uniform illumination would generate no complementary information.

The retrieved phase and amplitude of the gold structure is consistent with a reconstruction of the same specimen using far-field ptychography and inline-holography assuming a homogeneous object. The achieved resolution, as determined by the visibility of the Siemens star spokes in the phase image with diffuser, is between 100 and 200 nm, consistent with the limit given by the source size of 80 nm.

The complex-valued wavefronts retrieved simultaneously with the sample image are shown on the right hand side of Fig. 3. Propagation of these wavefronts to the detector plane is in good agreement with the flat field intensities. Unlike far-field ptychography, diversity in the dataset is localized in space: large areas where either *T* or *ψ* have no feature provide no coupling information. As a result, the illumination reconstruction is highly reproducible only around a central area of the field of view, where the sample provides sufficient mixing.

## Discussion

**Figure 4:**
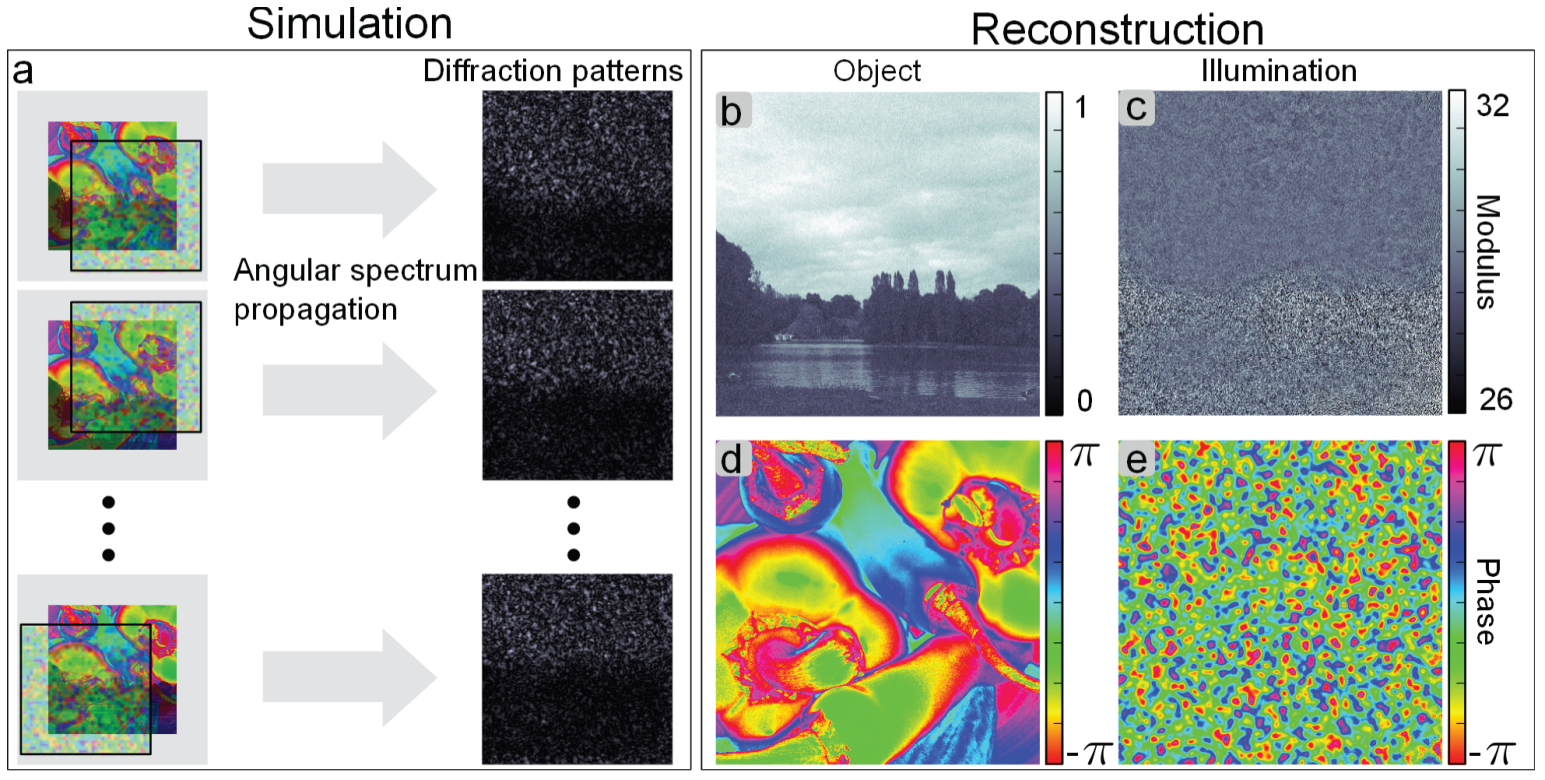
Simulation and reconstruction of a strong phase shifting and absorbing object. (a), the simulation scheme: diffraction data is generated by Fresnel propagation of the product of illumination function and phantom object for different relative positions. (b) and (c), reconstruction of the modulus of object and illumination function. (d) and (e), the corresponding phase.

We have validated with simulations that the principles introduced in this article are indeed not limited to the simple object used for our experimental demonstration. In particular, the sample can be a strong phase object, and no correlation between the phase and the magnitude of the transmission function is assumed. In the case depicted in Fig. 4, the phase and modulus of the simulated sample are generated from two different standard test images. The phase part of the transmission function covers the full 2*π* range, but the object is still assumed to be thin so that the propagation within the object can be neglected and the projection approximation holds. As illustrated in Fig. 4(a), diffraction data is simulated through near-field propagation of the product of the illumination and the sample transmission function for 16 different relative translations. Noise is simulated according to Poisson statistics for 1000 incident photons per pixel, a fluence similar to the one measured in the experiment. Wavelength, effective propagation distance and effective pixel size were the same as in the experiment. As shown on Fig. 4(b–e), the object and the illumination are successfully retrieved by our algorithm. The normalized root mean square error^37^ of the reconstructed object is 8% and the one of the illumination 10%.

In addition to diversity requirements, preparing the incident wave with a diffuser crucially eliminates the occurrence of so-called zero-crossings of the contrast transfer function^10^. In the past, the problems arising from the extinctions of specific spatial frequencies have been circumvented by combining measurements at multiple diffraction planes. One difficulty common to all near-field imaging techniques and not completely alleviated by our approach is the poor transfer of the lowest spatial frequencies in the image. In our simulations, smooth deviations from the ground truth are observed in the phase part of the reconstructed illumination and sample. Ways to reduce such artefacts experimentally or algorithmically are currently under investigation.

The effect of experimental parameters such as propagation distance, coherence, noise and scattering properties of the diffuser are currently under investigation as well. Preliminary calculations indicate that a poor choice of diffuser can slow down and even hinder convergence of the reconstruction algorithm. The diffuser that leads to optimal information mixing depends, among other things, on the effective pixel size. It should scatter at a fine enough scale to modulate strongly the interference of neighbouring pixels for different scanning positions. On the other hand, too fine structures create scattering that can not be sampled by the detector, thus adding an incoherent background that degrades the quality of the measurements.

Near-field ptychography will be used at its full potential when combined with tomography. The resulting tomograms are expected to exhibit fewer artefacts than currently existing holographic techniques since all wavefront imperfections can correctly be accounted for.

Besides imaging applications, the method may also be used as quantitative wavefront and optics characterization tool. When employed with other types of radiation, such as in visible light^38^ and electron holography, it could replace instances where through-focal series need to be acquired.

## Methods

### Experimental setup

The experiment was carried out at the nano-imaging endstation of beamline ID22 at the European Synchrotron Radiation Facility (ESRF), Grenoble, France. A schematic of the setup is shown in Fig. 1. The incoming X-ray wavefield had an energy of 16.9 keV with a 1.5% bandwidth and was focussed by a pair of Kirkpatrick-Baez mirrors (KB) to a virtual source of 80 nm. The detector, a FReLoN camera consisting of a scintillator, magnifying visible light optics and a CCD sensor was placed *z*_12_ = *z*_1_ + *z*_2_ = 530 mm downstream of the virtual source where *z*_1_ = 21.9 mm is the distance between source and sample and *z*_2_ = 508.1 mm is the distance between sample and detector. The dynamic range of the camera is 14 bit, the number of pixels is 2048^2^. Exposure time for a single diffraction pattern was 150 ms. The recorded diffraction patterns were corrected for dark current and rebinned by a factor of 2 resulting in diffraction patterns of a size of 1024^2^ pixels and an effective detector pixel size of Δ*s* = 1.512 μm. Propagation with the angular spectrum method yields a reconstructed pixel size in the sample plane given by the geometrical magnification *M*: 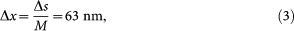
with 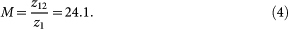
According to the Fresnel scaling theorem^33^, this cone beam geometry is equivalent to a parallel beam setup with an sample detector propagation distance of 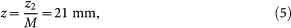
which, combined with the extent of the illumination, *W* = 64.5 μm, leads to an effective Fresnel number of 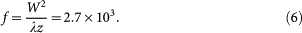
Additional distortions in the illuminating wavefront were optionally produced by placing the diffuser, a piece of paper, into the beam just upstream of the KB. The size of the illumination is defined by the opening of the entrance slits and extended the field of view slightly to avoid “diffraction artefacts” of these slits on the detector. From the slit opening the size of the illumination can be estimated to be about 76 μm at sample position (compared to 65.4 μm field of view at the sample position). The distance between the horizontal and vertical focal spot of the KB mirrors is of the order of 10 μm. The astigmatism resulting is negligible. It would anyway partly be covered by the reconstruction of the illumination.

The sample was a Siemens star test pattern with a height of about 700 nm electro-plated gold and a diameter of 30 μm. It was mounted on a high precision piezo-electric translation stage allowing movement transverse to the beam. The scanning displacements (depicted in Fig. 1 b) lie at the corner points of a large square with an edge length of 224 pixels or 14.112 μm. For each of these 4 corner points a sub-scan was performed on the corner grids of a small square with an edge length of 14 pixels or 0.882 μm so that in total 16 diffraction patterns where acquired.

### Reconstruction algorithm

The algorithm is based on the previously reported far-field difference map algorithm presented in Ref. 26. The reconstruction problem given by equation (1) can be reformulated as a search for the intersection of two constraint sets. The measured intensities define the modulus constraint and the overlap constraint is given by the known overlap of illumination and object for different scanning positions. The iterative reconstruction algorithm attempts to find the exit waves *χ*_j_ which are at the intersection of those two sets through projections of current estimates 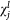 onto these sets, 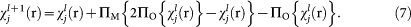
The modulus projector is commonly known in coherent diffractive imaging and is given by 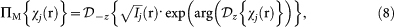
where the propagator 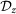 represent free-space propagation over a distance *z* as given by equation 2.

The overlap projector is defined as 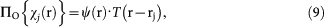
where *ψ*(**r**) and *T*(**r**) are the minimum of 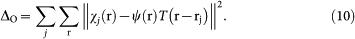
For the results presented here, the initial illumination was set equal to the square root of the intensity of one diffraction pattern and the initial object to a random modulus and phase distribution. The progress of the algorithm is monitored by the difference map error 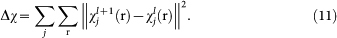
For the reconstructions presented here, a total number of 350 iterations (resp. 1000) was used for the dataset with (resp. without) diffuser.

Usually a residual phase ramp remains in the reconstructed object, an uncontrolled degree of freedom in the reconstruction. This phase ramp is evaluated and factored out of the reconstruction in a post-processing step.

## Author Contributions

M.S., P.C., B.E., M.D., I.Z., F.P. and P.T. conceived and designed the experiment. M.S., P.C., B.E., M.D. and P.T. performed the experiment. M.S. and P.T. analysed the data. M.S., P.C., B.E., M.D., I.Z., F.P. and P.T. contributed materials/analysis tools and wrote the paper.
